# Longitudinal choriocapillaris changes in the presence of reticular pseudodrusen

**DOI:** 10.1038/s41598-021-97771-w

**Published:** 2021-09-14

**Authors:** Christoph R. Clemens, Jost L. Lauermann, Boris Schmitz, Nicole Eter, Florian Alten

**Affiliations:** 1 Department of Ophthalmology, University of Muenster Medical Center, Domagkstrasse 15, 48149 Muenster, Germany; 2 Department of Rehabilitation Sciences, Faculty of Health, University of Witten/Herdecke, Witten, Germany; 3 Klinik Königsfeld der DRV, Center for Medical Rehabilitation, Ennepetal, Germany

**Keywords:** Macular degeneration, Retinal diseases

## Abstract

To determine longitudinal changes in choriocapillaris (CC) measures in eyes with reticular pseudodrusen (RPD) using optical coherence tomography angiography (OCTA). In this observational prospective study, 20 patients with exclusively RPD and no other alteration due to age-related macular degeneration were included. Eight RPD patients were re-examined at 5-year follow-up. Multimodal imaging was performed at baseline and at 5-year follow-up. OCTA CC images were analyzed for number, size and total area of flow deficits (FD), mean signal intensity, signal intensity standard deviation and kurtosis of signal intensity distribution in the ring area between a circle of 4 mm diameter and a circle of 6 mm diameter and in the superior ring quadrant. Area affected by RPD increased from 19.36 ± 8.39 mm^2^ at baseline to 37.77 ± 9.03 mm^2^ at 5-year follow-up. At baseline, percent of CC FD area was greater in RPD eyes (quadrant: p < 0.001; ring: p < 0.001) compared to controls. Besides, RPD eyes revealed a lower mean intensity signal (quadrant: p < 0.001; ring: p < 0.001). Evaluation of CC parameters suggested significant group × time interaction effects for CC FD (p = 0.04) and mean intensity signal (p = 0.004), in that RPD eyes presented increased CC FD and decreased mean intensity signal at follow-up. OCTA CC decorrelation signal further decreases in RPD patients over 5 years in both RPD-affected and RPD-unaffected macular areas.

## Introduction

Reticular pseudodrusen (RPD) represent an important risk factor for the development of late-stage age-related macular degeneration (AMD)^1^. Recently, the first use of multimodal imaging in a population-based cohort study of elderly individuals found a cumulative 5-year RPD incidence of 13.5%, which suggests a strong underestimation of this phenotype in fundus photography-based studies and emphasizes the relevance of RPD detection^2^. In 2010, the appearance of RPD was linked to accumulations of reflective material above the retinal pigment epithelium (RPE)—Bruch membrane band by Zweifel et al. using optical coherence tomography (OCT)^3^. Later, Greferath et al. reported the first clinicopathologic correlation of RPD in an eye imaged with OCT during lifetime confirming that RPD represent subretinal deposits that extend through the outer nuclear layer and compromise photoreceptor integrity^4^. Unlike RPD, soft drusen are lipid-rich extracellular material localized between the basal lamina of the RPE and the inner collagenous layer of Bruch’s membrane^5^. The exact pathophysiology of RPD formation and the progressing of outer retinal degeneration spatially associated with RPD lesions are currently subject of intensive research^6^.

Besides RPE dysfunction, flow impairment in the choriocapillaris (CC) is also discussed as a possible cause of RPD development, and in fact histologic studies showed CC ghost vessels in eyes with RPD^7,8^. With the availability of OCT angiography (OCTA), several groups demonstrated decreased flow signals at the CC level and increased areas of flow deficits in eyes with RPD^9,10^. Using OCT, mesopic and scotopic fundus-controlled perimetry, Sassmannshausen et al. demonstrated progressive outer retinal degeneration and corresponding impairment of photoreceptor function in eyes with RPD over 3 years^11^. Zhang et al. used adaptive optics scanning laser ophthalmoscopy over a 3.5-year period to describe the evolution of progression and regression of individual RPD lesions presumably reflecting the process of outer retinal atrophy^12,13^. So far, long-term OCTA data on CC changes in eyes with RPD are missing. In this context, the aim of this observational 5-year longitudinal study was to characterize CC changes in eyes with RPD using OCTA.

## Methods

### Demographics

In 2015, 20 RPD patients and 20 healthy age-matched control subjects without RPD or any other retinal pathology were prospectively recruited at the Department of Ophthalmology at the University of Muenster, Germany to quantitatively compare CC properties in OCTA. Cross-sectional baseline data showed decreased flow signals at the CC level in eyes with RPD as reported in detail in a previous report^9^. At baseline, only patients with distinct RPD in combined simultaneous confocal scanning ophthalmoscopy (cSLO) and spectral-domain optical coherence tomography (SD-OCT) imaging of the posterior pole in one eye were considered. Eyes were not eligible if any signs of soft drusen, macular neovascularization (MNV) or geographic atrophy (GA) due to AMD were observed in SD-OCT, cSLO, or fluorescein angiography as reported elsewhere^9^.

For the present longitudinal analysis, participants were recalled at 5-year follow-up. Axial length (AL) was measured with a non-contact partial coherence laser interferometry (IOL Master 500, Carl Zeiss Meditec, Jena, Germany) and best-corrected visual acuity (BCVA) was measured. All investigations were performed in accordance with the declaration of Helsinki and after the approval of the ethical committee of the medical association Westfalen-Lippe and the Westphalian Wilhelms-University of Muenster (project-no. 2014-413-f-S).

### Imaging and image analysis

Subjects had been at rest before OCTA images were recorded and the consumption of neither caffeine nor nicotine was allowed 2 h prior to OCTA. According to baseline examination, imaging was conducted with a commercial spectral domain OCT-system (AngioVue, RTVue XR Avanti SD-OCT, Optovue, Fremont, CA, USA). Images were recorded by an experienced operator under mesopic lighting conditions. The device delivered volumetric scans of 300 × 300 A-scans at 70,000 A-scans per second using a light source at 840 nm. Two consecutive B-scans covering the central 6 × 6 mm^2^ field were done to compute inter-B-scan decorrelation. CC analysis was performed using the manufacturer’s automated segmentation of 10 μm above to 30 μm below Bruch’s membrane. The proprietary software (AngioVue Analytics software 2017.1.0.151) includes automated segmentation, an eye tracking function, and an artefact removal function. For quality control, images showing inadequate signal (signal quality index [SQI] < 7) or an OCTA motion artifact score of three or four were excluded. The accuracy of CC segmentation was checked and images were excluded if segmentation was incorrect^14^. Baseline and 5-year follow-up CC OCTA en-face image data were exported, and a grid was applied to the image using Adobe Photoshop CS6 (Adobe Systems, CA, USA). The grid was placed automatically and the correct alignment of the grid center with the fovea was checked.

**Figure 1:**
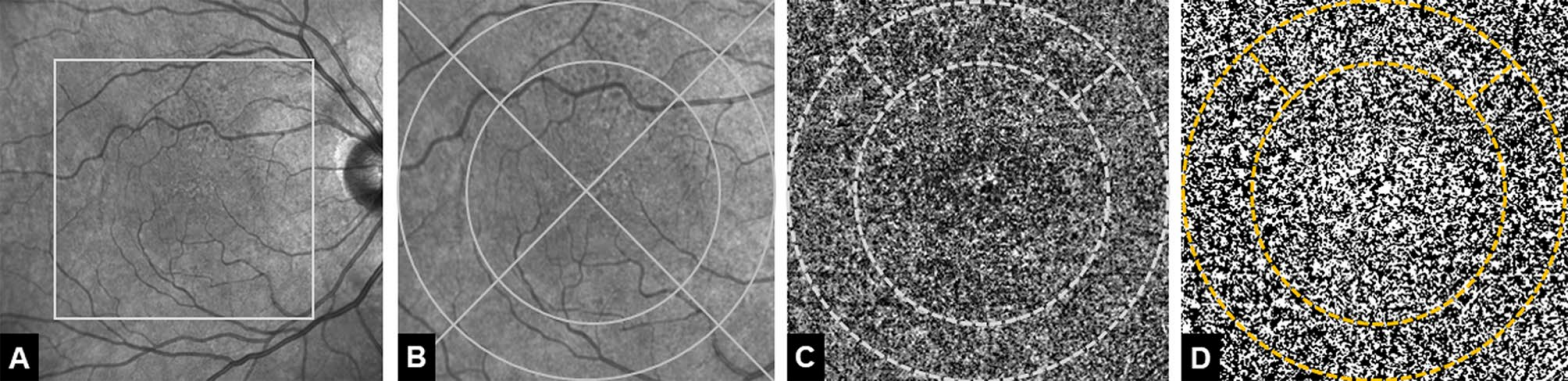
Representative patient with reticular pseudodrusen (RPD) at baseline. (**A**) Near-infrared confocal scanning laser opththalmoscopy (cSLO) image showing hyporeflective dots located superiorly to the fovea. Rectangle marks the area of 6 × 6 mm magnified in (**B**) Overlaid grid with two circles of 4 mm and 6 mm diameter as well as two lines dividing the area in four quadrants. (**C**) 6 × 6 mm optical coherence tomography angiography choriocapillaris image. Choriocapillaris layer segmentation extending from 10 μm above to 30 μm beneath the Bruch’s membrane. Dotted line marks the 4-mm-diameter, 6-mm-diameter circles centered on the fovea and the superior quadrant. (**D**) Image after automatic local thresholding done with the Phansalkar method, flow deficits are shown in white.

Two aspects were important in defining grid dimensions. First, the 3 mm ring measure of the ETDRS grid as standardized tool for evaluating AMD hypotheses could not be applied because part of the RPD patients expectedly developed late-stage AMD during follow-up resulting in significant CC alterations beyond the 3 mm ring area^15,16^. Thus, the grid had to be adjusted and a ring of a 4 mm diameter circle and a 6 mm diameter circle was chosen to perform CC analysis. Secondly, the initial development of RPD usually begins perifoveal superior^17^. Correspondingly, in our RPD study group all patients showed RPD lesions in the superior quadrant at baseline while the rest of the macula was mostly unaffected. Therefore, the ring was divided into quadrants and we performed the CC analysis (1) within the superior ring quadrant and (2) within the ring (Fig. 1).

CC flow characteristics in OCTA were quantified according to a method previously presented by Spaide^18^. Briefly, automatic local thresholding was performed using the Phansalkar method in Fiji (an expanded version of ImageJ version 1.51a, available at fiji.sc) in consideration of recently presented recommendations on OCTA CC image analysis^19,20^. The “Analyze Particles” command of Fiji was used to count flow deficit (FD) areas and measure the size of each FD area to determine the total size of FD [%]. In accordance with previous studies, FD areas smaller than 24 µm were removed as they appear not to be physiologically relevant and are more likely to correspond to noise^21^. Data of each eye was logarithmically binned and fitted with a linear regression line that followed the equation *log*10(*number*) = m*log10(*area*) + *c*. Slope (m) and Y-Intercept (c) as well as total size of FD were statistically analyzed. In addition, CC images were evaluated for mean signal intensity, standard deviation of mean signal intensity and kurtosis of signal intensity distribution. Kurtosis is a dimensionless statistical value to quantify the non-Gaussianity of a distribution and was used to characterize the signal intensity distribution in the grey level CC images.

cSLO near-infrared imaging (IR, λ = 830 nm) (Spectralis, Heidelberg Engineering, Germany) was performed with a minimum resolution of 768 × 768 pixels. The field of view was set at 30° × 30° and centered on the fovea. SD-OCT volume scans (high-speed mode, 30° × 25°, ART minimum nine frames, 61 B-scan) were obtained. cSLO images and SD-OCT scans were used to determine presence of RPD in the study group and the integrity of all retinal layers in the healthy control subjects. RPD were defined as present only if they were identified in both near-infrared images and SD-OCT scans. Characteristics of RPD lesions in different imaging modalities have been reported in detail elsewhere^22^. The total area affected by RPD was outlined in cSLO IR images at baseline and at 5-year follow-up as described elsewhere^23^.

According to recent consensus definitions, follow-up OCT scans were analyzed concerning the development of atrophy in areas affected by RPD at baseline^24,25^. Atrophy was graded as complete RPE and outer retinal atrophy (cRORA), incomplete RPE and outer retinal atrophy (iRORA), complete outer retinal atrophy (cORA), and incomplete outer retinal atrophy (iORA)^24,25^.

### Statistical analysis

Statistical analyses were performed with R (R: A Language and Environment for Statistical Computing, R Core Team, R Foundation for Statistical Computing, Vienna, Austria, 2016) and Prism 9.0 (GraphPad Software nc., La Jolla, USA). Constant variables are expressed as mean ± standard deviation (SD). Categorical variables are presented as n (%). Data were tested for normal distribution using Kolmogorov–Smirnov test. Between-group differences from baseline were analysed using repeated measures two-way ANOVA (for time × group interactions). Within-group time effect was reported if no between-group differences were detected. Differences at baseline were assessed using two-sided unpaired t-test. Linear regression slopes and intercepts were compared as described reporting p and F values derived from F tests^26^. Mean signal intensity distribution in the grey level CC images was fitted using sixth order polynomial nonlinear regression. Significance was accepted for p < 0.05.

### Ethical approval

All investigative procedures were conducted in accordance with the tenets of the Declaration of Helsinki, and the study was approved by the local ethical committee of the University of Muenster.

### Informed consent

Informed consent was obtained from all participants included in the study.

## Results

Eight eyes of eight RPD patients and eight eyes of eight control subjects were re-examined at 5-year follow-up. Further follow-up data of 12 patients and 12 controls were not available because subjects were no longer willing or able (because of general illness or death) to further participate in the study.

**Figure 2:**
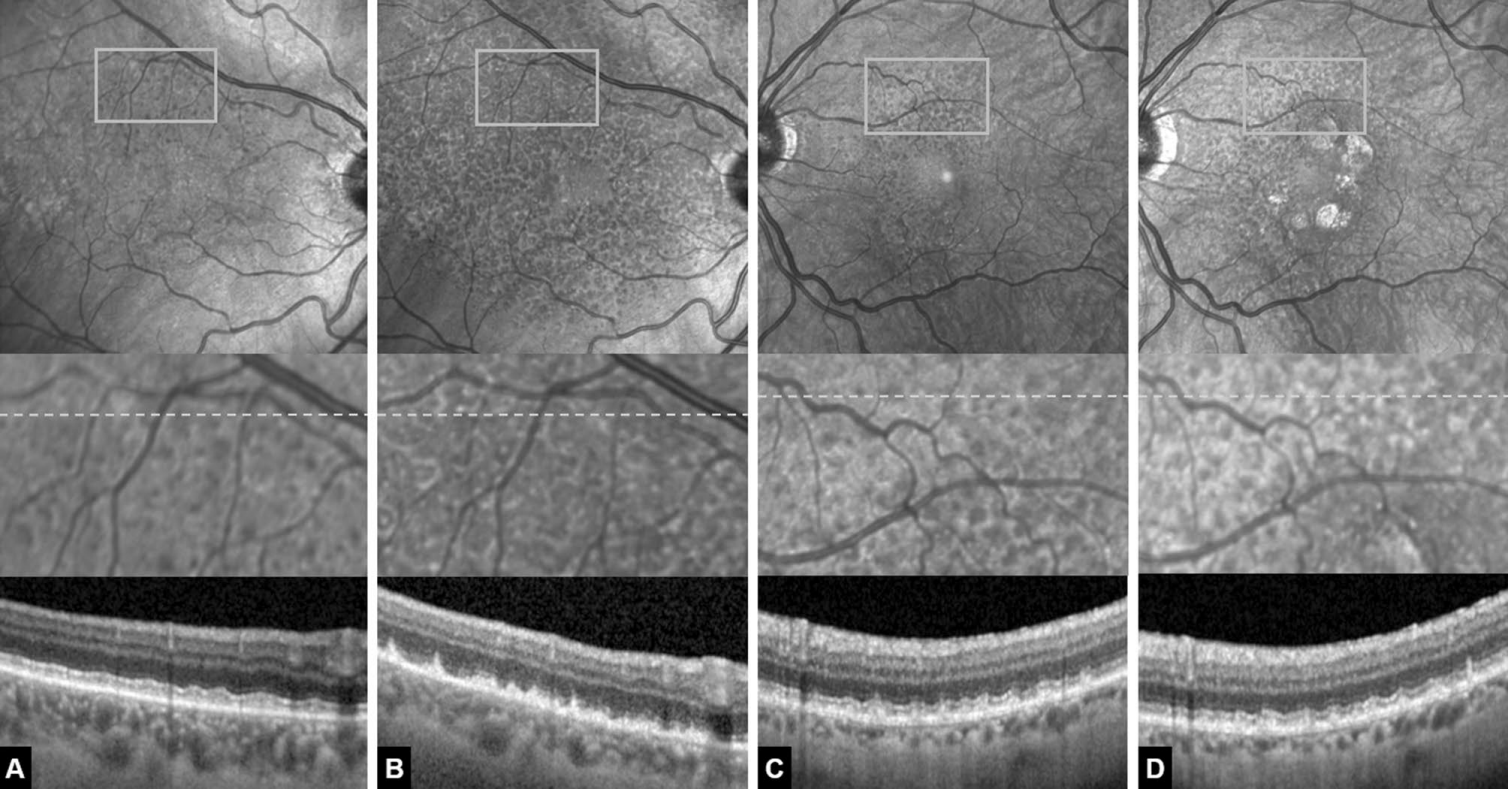
(**A**, **B**) Example of a 70-year-old female reticular pseudodrusen (RPD) patient that developed a significant increase in RPD-affected area over the 5-year follow-up yet no late-stage form of age-related macular degeneration. (**A**) Baseline. Above near-infrared confocal scanning laser ophthalmoscopy (cSLO) image showing hyporeflective dots superiorly located to the fovea. Rectangle marks the area magnified below. Grey dotted line marks the location of the optical coherence tomography (OCT) scan below. (**B**) Same eye after 5 years. Note the increase in RPD-affected area and the increase in lesion density visible both in the cSLO en-face image above as well as in the magnified image. The OCT scans reveal early RPD stages at baseline and predominantly stage-three RPD lesions at 5-year follow-up. Also note signs of incomplete outer retinal atrophy in (**B**). There is detritus on the intact RPE monolayer with remnants of the ellipsoid zone but no hypertransmission. Individual RPD lesions are harder to discern. (**C**, **D**) Example of a 71-year-old female RPD patient that developed geographic atrophy over the 5-year follow-up. (**C**) Baseline. (**D**) Same eye after 5 years. Note the perifoveal patches of geographic atrophy and a moderate increase in RPD-affected area and lesion density. The OCT scan appears rather similar to the baseline image without any signs of outer retinal atrophy.

Participants’ characteristics at baseline and follow-up are presented in Table 1. At baseline, groups were age-matched (p = 0.69)^9^. However, the dropout of 12 patients and 12 controls created an imbalance and led to a significant age difference at the 5-year follow-up (p = 0.01). OCTA CC data are shown in Table 2. The absolute retinal area affected by RPD increased by 95% from 19.36 ± 8.39 mm^2^ at baseline to 37.77 ± 9.03 mm^2^ at 5-year follow-up. During 5-year follow-up, three RPD patients did not develop late-stage AMD, one patient developed perifoveal GA (cRORA), and four had to be treated with 9.25 ± 4.9 anti-VEGF injections due to foveal MNV development. In those patients, neither GA nor MNV lesions affected the analysed ring area between the 4 mm and 6 mm circle. At 5-year follow-up, iORA was present in three eyes showing a loss of the ellipsoid zone band in areas that were RPD-affected at baseline (Fig. 2). None of the control patients developed a retinal pathology during follow-up. All OCT-A scans had an adequate signal strength (> 7) and did not show excessive motion error. All OCTA images were graded as having a motion artifact score of 1 or 2 and all showed accurate segmentation of the CC.

**Table 1:** Participants’ anthropometric data.

	Healthy controls (n = 8)	RPD patients (n = 8)
Age (years)	60.8 ± 9.1	73.1 ± 5.7
Follow-up (months)	58.9 ± 1.3	60.4 ± 2.1
Axial length (mm)	23.56 ± 0.7	23.56 ± 0.3
BCVA(LogMAR; [Snellen]) baseline	0.0 ± 0.0 (6/6)	0.0 ± 0.0 (6/6)
BCVA(LogMAR; [Snellen]) follow-up	0.0 ± 0.0 (6/6)	0.32 ± 0.2 (6/12)
Late-stage AMD at follow-up	n/a	5 (62.5%)

Data are means ± SD or n (%).

*BCVA* best corrected visual acuity.

**Table 2:** Choriocapillaris parameters at baseline and follow-up.

	RPD patients		Healthy controls	
	Baseline	5-year follow-up	Baseline	5-year follow-up
**Superior quadrant**				
CC flow deficits (%)	38.36 ± 4.34^a^	45.48 ± 3.54^b^	33.34 ± 2.29	35.83 ± 2.52
Intensity mean	103.21 ± 8.66^a^	88.32 ± 5.47^c^	117.33 ± 5.71	113.80 ± 6.30
Intensity SD	56.50 ± 3.09^a^	56.56 ± 6.41	53.84 ± 1.82	52.78 ± 4.34
Kurtosis	1.67 ± 0.11	1.63 ± 0.10	1.69 ± 0.08	1.69 ± 0.07
**Ring**				
CC flow deficits (%)	36.39 ± 4.10^a^	43.44 ± 2.80^c^	32.29 ± 2.08	34.81 ± 3.12
Intensity mean	103.63 ± 8.47^a^	86.71 ± 3.51^c^	117.37 ± 5.73	114.12 ± 6.09
Intensity SD	53.31 ± 2.50	52.44 ± 5.14	52.75 ± 1.15	53.48 ± 1.90
Kurtosis	1.62 ± 0.09^a^	1.53 ± 0.07^c^	1.64 ± 0.03	1.67 ± 0.07

^a^Significantly different at baseline.

^b^Significantly different from baseline by two-way repeated measures ANOVA (time effect).

^c^Significant between-group difference by two-way repeated measures ANOVA (interaction effect). Data are mean ± SD. Data were tested for normal distribution using Kolmogorov–Smirnov test. *CC* choriocapillaris, *SD* standard deviation, *RPD* reticular pseudodrusen.

### Differences in CC parameters between controls and RPD patients at baseline

**Figure 3:**
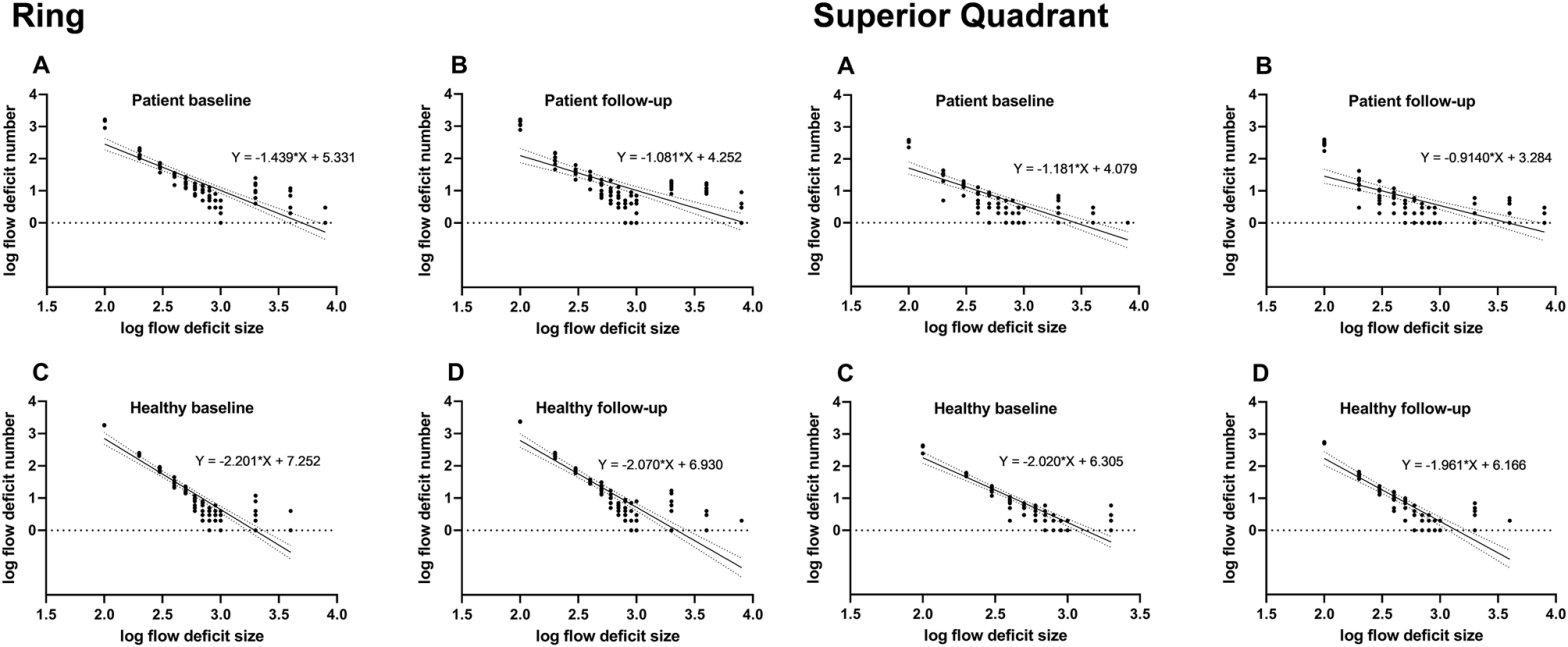
Baseline to follow-up changes of flow deficits shown by log–log plot with logarithmic binning in the ring area (above) and in the superior ring quadrant (below). Individual data points are shown with linear regression and 95% confidence Interval. The respective equations are given with slope (m-values) and Y-intercept (c-value).

At baseline, percentage of CC FD area was greater in RPD eyes (quadrant: p < 0.001; ring: p < 0.001) compared to controls. FD analysis also revealed an altered distribution of FD regions based on a lower y-intercept and flatter slope (difference between slopes; quadrant: p < 0.0001, F = 24.99, ring: p < 0.0001, F = 26.13) in RPD eyes (Figs. 3/4), i.e. RPD eyes showed multiple confluent FD regions, while healthy controls exhibited a higher proportion of smaller, non-confluent FD regions.

**Figure 4:**
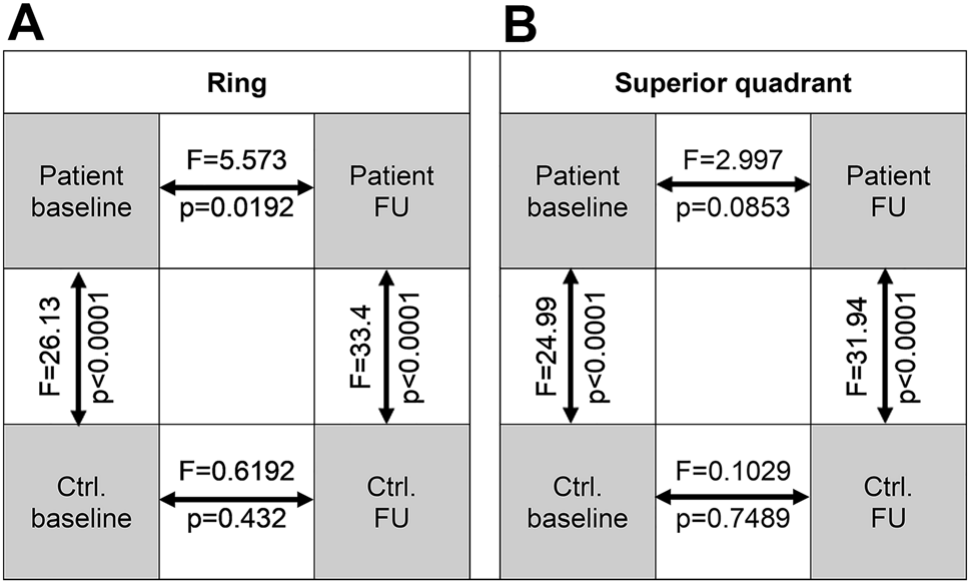
Comparison matrix of flow deficit linear regression slopes for (**A**) the ring area and (**B**) the superior ring quadrant. Arrows indicate respective comparisons with p and F values (derived from F tests). *Ctrl* control, *FU* follow-up.

**Figure 5:**
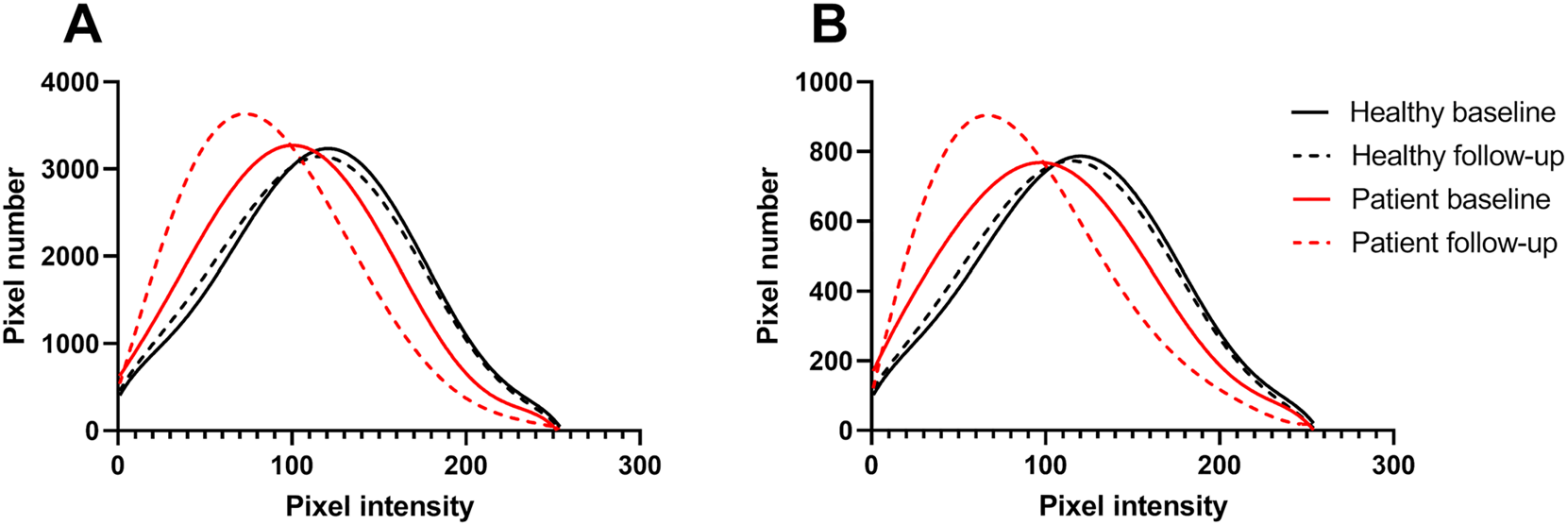
Plots illustrating the distribution of frequencies of grey level intensities in (**A**) the ring area and (**B**) the superior ring quadrant of patients and controls at baseline and 5-year follow-up. Mean pixel intensity distribution in healthy controls (n = 8) was identical at baseline and follow-up. Mean pixel intensity distribution in patients (n = 8) was non-identical at baseline and follow-up and different to patients’ distribution at both time points. Individual data points were fitted by sixth order polynomial nonlinear regression.

In addition, the quantitative analysis of the CC revealed a significantly lower mean intensity signal (quadrant: p < 0.001; ring: p < 0.001) and a higher standard deviation of signal intensity (quadrant: p = 0.04; ring: p = 0.82) in RPD eyes compared to healthy individuals, i.e. a lower and more heterogeneous OCTA signal in RPD patients (Table 2) (Fig. 5).

### Differences in CC parameter alterations after 5 years

Evaluation of CC parameters in the ring area suggested significant group × time interaction effects for CC FD (p = 0.04) and mean intensity signal (p = 0.004), in that RPD eyes presented increased CC FD and decreased mean intensity signal at follow-up (Table 2) (Fig. 5). In the superior quadrant, significant group × time interaction effects were only found for mean intensity signal (p = 0.02), not for CC FD (p = 0.06).

Comparison of linear regression lines revealed that slopes of FD distribution in RPD patients were different at baseline and follow-up (difference between slopes; quadrant: p < 0.0853, F = 2.997, ring: p < 0.0192, F = 5.573) (Figs. 3/4). In contrast, comparison of linear regression lines in healthy individuals revealed that slopes were not significantly different at baseline and follow-up (difference between slopes; quadrant: p = 0.7489, F = 0.1029, ring: p < 0.432, F = 0.6192) (Figs. 3/4).

## Discussion

In this study, we evaluated longitudinal CC changes in the presence of RPD using OCTA. In brief, we found that (1) CC decorrelation signal is significantly reduced in RPD patients (2) CC decorrelation signal further decreases in RPD patients over 5 years (3) CC alterations in RPD eyes can be observed in both RPD-affected and RPD-unaffected macular regions.

With the availability of OCTA, several groups studied CC perfusion in RPD patients using different OCTA image analysis approaches. Uniformly, the data indicated an impaired CC decorrelation signal in RPD-affected eyes^9,10^. In accordance with those previous studies, our data shows higher FD values and lower flow signal values in the RPD group at baseline compared to healthy controls. So far, there is no longitudinal CC data available based on OCTA.

The main finding of our study is a further deterioration of already impaired CC decorrelation signal in RPD eyes over time, which may reflect pathophysiologic processes in the context of atrophy development. In 2013, Spaide introduced the term outer retinal atrophy (ORA) as a new form of late-stage AMD distinct from the late-stage form of GA^13,24^. ORA follows RPD regression including outer nuclear layer collapse, a poorly visible or non-visible ellipsoid zone, and a thin choroid, often with an apparently intact RPE layer^13^. Zhang et al. were able to replicate Spaide’s original findings in a longitudinal 3.5-year study based on multimodal imaging including adaptive optics scanning laser ophthalmoscopy describing distinct lifecycles of individual RPD lesions with initial growth and subsequent regression. In the process of RPD regression, the authors report an almost complete loss of the outer retinal architecture^12^.

Another important aspect of our study to be addressed is the potential influence of anti-VEGF on the CC perfusion properties as half of the RPD patients received intravitreal injections due to conversion to neovascular AMD during follow-up. To date, there is no definite consensus on the temporary and permanent changes in CC perfusion induced by anti-VEGF therapy in neovascular AMD eyes. However, the suppression of VEGF is demonstrably associated with a reduction of CC endothelial cell fenestrations^27^. Besides, nAMD eyes with a long-term history of anti-VEGF therapy were characterized by decreased vascular densities in the CC measured in OCTA^28^. And notably, the finding of ‘macular atrophy’, a term describing atrophy developing under anti-VEGF therapy, has been a growing concern in that anti-VEGF therapy itself may contribute to the development of new atrophy of the outer retina^29^. On the other hand, a decreased CC vascularity may also be secondary to RPE degeneration caused by AMD pathophysiology itself. Since the RPE produces a variety of growth factors that maintain CC, impairment of the RPE inevitably damages the CC vasculature. The question of whether anti-VEGF plays a role in the decorrelation signal decrease in RPD patients over time will remain difficult to answer because the proportion of RPD eyes that develop MNV over time and require treatment is high.

Like soft drusen, RPD could produce projection artifacts, and thereby confound CC measurements in that the increase in RPD-affected area causes more FD and less signal intensity compared to baseline^30^. However, using a point-to-point correlation, Nesper et al. found that RPD identified on OCT B-scans and structural OCT slabs showed no shadow artifacts^10^.

In our analysis, healthy controls also revealed higher FD values and lower flow signal values at 5-year follow-up compared to baseline. This finding is in accordance with histologic data reported by Ramrattan et al. who showed that histologically normal maculae decreased in density and diameter of CC vessels with advancing age^31^. Correspondingly, increasing amounts of FD were found on both 3 × 3-mm and 6 × 6-mm OCTA CC images in normal aging eyes^32^.

Interestingly, CC decorrelation signal significantly decreases in RPD patients over time both in the superior quadrant that was already RPD-affected at baseline and in the complete ring area that was largely RPD-unaffected. This observation supports the notion that the development of RPD rather reflects a compromised state of the entire macula and is less an expression of a delimited localized event. Further, one might argue that CC changes precede the development of RPD and that changes in the CC are not secondary to RPD development. Notably, the parameter CC FD has recently received increasing attention, as a higher CC FD was independently associated with a higher risk for progression to atrophy. Thus, CC FD was proposed as prognostic biomarker for enhancing risk stratification and prognostication of patients with intermediate AMD and for evaluating the risk for progression of GA^33^.

Due to the small number of patients, we did not perform further subgroup analysis. Future long-term RPD studies should consider CC differences at baseline between RPD eyes that develop late-stage AMD at follow-up visits and RPD eyes that do not.

The quantitative evaluation of OCTA CC images was recently the subject of debate. In this context, binarization represents a crucial step in current OCTA CC image analysis using thresholding algorithms to convert grayscale pixel values into binary pixel values representing non-flow and flow, respectively. Laiginhas et al. recently postulated that local thresholding strategies, such as Phansalkar or others, are significantly superior to global ones, such as Otsu or others, for quantifying CC and should be preferred^34^. However, direct comparisons between different strategies should not be performed^35^. So far, there has been no consensus on binarization practice and numerous studies have shown that different binarization methods resulted in significantly different FD measures.

In addition to binarization using thresholding algorithms, compensation strategies and CC slab localization are the two other most relevant topics in quantitative analysis of OCTA CC images that are currently under intense discussion^36,37^. In this study, the automatic default setting of 10 μm above to 30 μm below Bruch’s membrane was used in line with numerous previous studies. Various slabs with varying offsets and thicknesses have been used and so far, no consensus has emerged about the optimal position and thickness of the CC slab^38^. Thus, there is an unmet need for a uniformly accepted strategy to quantify CC in future studies^34^.

Obviously, the number of included patients precludes any definitive interpretation. Yet, the inclusion of pure RPD eyes at baseline and a follow-up of 5 years both represent important strengths of this study. Since the begin of the study 5 years ago, there has been significant improvement in OCTA imaging of the CC that could not be considered in this study such as OCTA CC image averaging or the use of swept source OCTA. Updates of the AngioVue Analytics software may have influenced the measures at baseline and 5-year follow-up. Besides, FD analysis of the CC varies according to the device used and the post processing methods. Thus, results may not be transferred to other OCTA devices. Finally, there is an age difference between the groups. The dropout of 12 patients and 12 controls led to a significant age difference at the 5-year follow-up, which represents a limitation. Nevertheless, this age difference seems to be negligible in view of the data of Zheng et al., who performed a large study including normal subjects of all age groups and found a general dependence of CC FD on age in healthy subjects. However, this study’s data do not show a relevant difference when looking at the specific age groups and specific topographic measurements at issue in our study. In particular, the study showed that the age-dependent increase in CC FD [%] is largely driven by the central 1-mm area, which was not included in our grid^32^.

In conclusion, we found that CC decorrelation signal significantly decreases in RPD patients over time, which was measurable in both RPD-affected and RPD-unaffected macular regions. These observations may reflect alterations in the context of atrophy development in RPD eyes.

## Data Availability

Data are available on request.
